# Hepatic toxicology following single and multiple exposure of engineered nanomaterials utilising a novel primary human 3D liver microtissue model

**DOI:** 10.1186/s12989-014-0056-2

**Published:** 2014-10-20

**Authors:** Ali Kermanizadeh, Mille Løhr, Martin Roursgaard, Simon Messner, Patrina Gunness, Jens M Kelm, Peter Møller, Vicki Stone, Steffen Loft

**Affiliations:** Department of Public Health, University of Copenhagen, Section of Environmental Health, Copenhagen, 1014 Denmark; Heriot Watt University, School of Life Sciences, Nanosafety research group, Edinburgh, EH14 4AS UK; InSphero AG, Wagistrasse 27, Schlieren, 8952 Switzerland

**Keywords:** 3D human liver microtissue, Engineered nanomaterials, Inflammation, Lipid peroxidation, Genotoxicity

## Abstract

**Background:**

The liver has a crucial role in metabolic homeostasis as well as being the principal detoxification centre of the body, removing xenobiotics and waste products which could potentially include some nanomaterials (NM). With the ever increasing public and occupational exposure associated with accumulative production of nanomaterials, there is an urgent need to consider the possibility of detrimental health consequences of engineered NM exposure. It has been shown that exposure via inhalation, intratracheal instillation or ingestion can result in NM translocation to the liver. Traditional *in vitro* or *ex vivo* hepatic nanotoxicology models are often limiting and/or troublesome (i.e. reduced metabolism enzymes, lacking important cell populations, unstable with very high variability, etc.).

**Methods:**

In order to rectify these issues and for the very first time we have utilised a 3D human liver microtissue model to investigate the toxicological effects associated with a single or multiple exposure of a panel of engineered NMs (Ag, ZnO, MWCNT and a positively charged TiO_2_).

**Results:**

Here we demonstrate that the repeated exposure of the NMs is more damaging to the liver tissue as in comparison to a single exposure with the adverse effects more significant following treatment with the Ag and ZnO as compared with the TiO_2_ and MWCNT NMs (in terms of cytotoxicity, cytokine secretion, lipid peroxidation and genotoxicity).

**Conclusions:**

Overall, this study demonstrates that the human microtissue model utilised herein is an excellent candidate for replacement of traditional *in vitro* single cell hepatic models and further progression of liver nanotoxicology.

## Background

The rapid expansion of technological, scientific and commercial uses of molecular scale materials, their assembly and their unique properties has led to an escalating interest in the fields of nanoscience, nanotechnology and nanomedicine [1]. There are now over 1600 consumer products on the market with a claim of containing aspects of nanotechnology [2]. These applications include medicine, electronics, engineering, cosmetics and textiles [3]. As a negative however, the same unique chemical and physical characteristics which make NMs so desirable might also contribute to their potential adverse effects, which raises concerns that some NMs could be hazardous for people living and working with said materials [4,5]. The small size of particulate nanomaterials results in a high surface area to volume ratio, which potentially offers a greater biological activity per given mass compared to larger-size materials [6]. In addition to this, the surface reactivity per unit surface area can be greater at the nano-scale due to higher curvature of the surface [7]. As the potential for public and occupational exposure is likely to rise with increasing production of nanomaterials, there is an urgent need to consider the possibility of detrimental health consequences of engineered NMs of all kinds. The health risk is assessed based upon the level of exposure to the NM, toxicity of the material in question, route of exposure and the persistence of the particular material in the organism.

The lungs and the gastrointestinal tract are in constant contact with the external environment, hence these systems are the primary exposure sites for NMs [5,8]. However it has been demonstrated that some NMs can translocate from these primary exposure sites [8,9]. As a secondary exposure site the liver is extremely important, as it has been shown to accumulate NMs at much higher concentrations compared to other organs [8-11]. In addition, the prominent advances in nanomedicine will result in an increase in direct entry of NMs into the circulatory system. The presence of NMs in the blood will result in the materials reaching the liver faster and in large quantities and will cause the induction of an immune response from the organ [12-15].

The liver has a crucial role in metabolic homeostasis. It is responsible for the storage, synthesis, metabolism and/or redistribution of carbohydrates and fats [16]. The organ produces large numbers of serum proteins, an array of enzymes and acute phase proteins. It is also the principal detoxification centre of the body, removing foreign substances and waste products [16]. The liver is characterised by its distinct populations of cells, each with their own unique morphology amongst which the hepatocytes, the resident macrophage population (Kupffer cells - KCs) and sinusoidal endothelial cells (SECs) being of utmost importance due to their numbers, location within a healthy liver and the wide arrays of functions that are crucial in overall liver activity [13,14,16-19].

It is widely argued that *in vitro* hepatocyte systems (including cell lines and systems without optimal artificial extracellular matrices) might not be ideal for functional and metabolic studies as the cells lose key liver specific functions especially their cytochrome P450 (CYP450) activity [18,20,21]. Hence the cells used in such experiments are not representative of “real” hepatocytes *in vivo*. In addition, most hepatic studies of the effects of nanomaterials are undertaken on hepatocyte cell lines or primary cultures lacking any non-parenchymal cells. However these cells are extremely important as they are components of the reticulo-endothelial system and are involved in the clearance of particles from blood as well as being crucial in the initiation of an immune response to foreign material challenge. Finally, in typical *in vitro* or *ex-vivo* systems cells have a very limited life span, therefore the opportunity for repeated exposure is not usually possible. To address all above issues for the very first time in a study of its kind we have utilized the InSphero 3D human liver microtissue model generated from primary human hepatocytes and liver-derived non-parenchymal cells [22,23] in order to investigate the adverse effects of a panel of engineered NMs as a model of the human liver.

The panel of NMs chosen for this study were part of the Joint Research Centre (JRC) repository for nanomaterials. These materials included a zinc oxide (ZnO), a silver (Ag), a multi-walled carbon nanotube (MWCNT) and a positively charged titanium dioxide (TiO_2_), which are currently incorporated in numerous products including sunscreens, cosmetics, sporting goods and anti-bacterials [2].

This study therefore investigated the toxicological properties of the panel of four engineered nanomaterials on the human liver microtissue following a single or multiple (five) exposures. Here the materials and the tissue were fully characterised before NM induced cell cytotoxicity, inflammatory response (cytokine secretion), oxidative stress (lipid peroxidation), DNA damage and hepatic function (albumin production) were investigated.

## Results

### Characteristics of pristine and dispersed nanomaterials

The investigated NMs were extensively characterised by a combination of analytical techniques in order to infer primary physical and chemical properties useful to understand their toxicological behaviour. A list of the measured physical and chemical properties of selected nanomaterials was previously described and has been reproduced in Table 1 [24]. In order to investigate how the NM characteristics are affected in human liver maintenance medium, the hydrodynamic size distributions of the NMs was measured after dispersion in the medium (5-20 μg/ml range) (Table 1).

**Table 1:** **Main physical and chemical properties of tested NMs (adapted and reproduced from 24)**

**NM code**	**NM type**	**Phase**	**XRD size [nm]**	**TEM size**	**Surface area (BET)** **[m** ^**2**^ **/g]**	**Known coating**	**Size in human liver maintenance medium (Nanosight)** ^**Ψ**^	**Dissolution in medium (%)**
NM 110	ZnO	Zincite	70 to >100	20-250/50-350	14	None	130 ± 48 (64%)	23.36-46.70%
							242 ± 32 (25%)	
NM 300	Ag	Ag	7^$^	8-47 (av. 17.5)	NA	PVP capped	98 ± 43	<1%
			14^£^					
			<18					
NM 400	MWCNT	-	-	D: 5-35	298	None	NA	NA
				L: 700-3000				
NRCWE 002	TiO_2_	Rutile	10	80-400	84	Triethylpropylaminosaline	278 ± 151	NA

^$^wet XRD in capillary tube.

^£^dried samples.

^Ψ^intensity based size average in biological media within 30 mins.

*Abbreviations*: *D* Diameter, *L* Length, *XRD* X-ray diffraction.

### Microtissue characterisation

**Figure 1:**
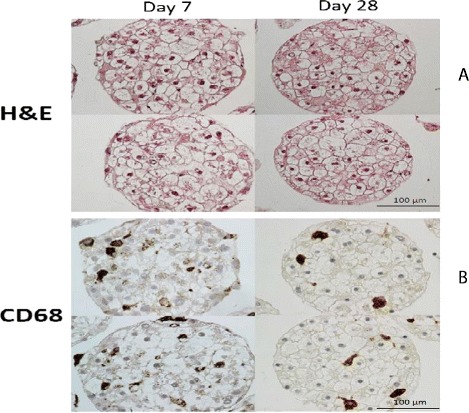
**Morphological characterization of intact 3D human liver microtissue. A)** H&E staining **B)** CD68 immunohistochemistry of formalin fixed paraffin embedded microtissues after 7 and 28 days in culture. Two representative tissues are shown per time point (magnification 200×).

In order to evaluate the applicability of heterotypic 3D human liver microtissues for prolonged, repeated nanomaterial exposure, the morphological characterization of the microtissues was performed. To investigate the 3D cytoarchictecture and the histological phenotype, the human liver microtissues were stained with Hemalaun and Eosin (H&E) (Figure 1A). The H&E staining indicates the presence of typcial liver histotypic features, such as a cuboidal hepatocyte cell shape with tight cell-cell contacts. These features are preserved over the tested culture period. The microtissues were 7 days old at first treatment time (0 hr in Table 2). The data presented here is representative of span of 21 days demonstrating healthy tissues over the whole assay period. The absence of a necrotic core suggests that sufficient oxygen and nutrients are transported into the inner regions of the microtissue. Since the primary hepatocytes are co-cultured with primary non-parenchymal cells, the presence of KC was tested with macrophage specific marker CD68 (Figure 1B). The immunohistochemistry analysis shows that KCs are indeed incorporated into the microtissues and present troughout the tested culture period. In addtion some KCs were found to be loosly attached to the microtissue. The cells show a typical dynamic morphology with both elongated and rounded cell shapes. Here we were unable to identify significant numbers of SECs in the selected microtissues following the incorporation of the particular batch of non paranchmal cells.

**Table 2:** **Microtissue maintenance and NM treatment in the singular and repeated exposure experiments over a period of 15 days**

**Single exposure**	**End-points investigated**
Treatment **1** – 0 hr (**day 0**)	-
Change medium – 24 hr (**day 1**)	AK assay, cytokine secretion, albumin
Change medium – 72 hr (**day 3**)	Albumin
Change medium – 168 hr (**day 7**)	AK assay, cytokine secretion
Change medium – 216 hr (**day 9**)	Albumin
Change medium – 264 hr (**day 11**)	-
Change medium – 336 hr (**day 14**)	-
Change medium – 360 hr (**day 15**)	AK assay, cytokine secretion, lipid peroxidation, DNA damage, albumin
**Multiple exposure**	**End-points investigated**
Treatment **1** – 0 hr (**day 0**)	-
Change medium – 24 hr (**day 1**) – 48 hr recovery period	AK assay, cytokine secretion
Treatment **2** – 72 hr (**day 3**)	AK assay, cytokine secretion, albumin
Change medium – 96 hr (**day 4**) - 48 hr recovery period	AK assay, cytokine secretion, albumin
Treatment **3** – 144 hr (**day 6**)	AK assay, cytokine secretion
Change medium – 168 hr (**day 7**) - 48 hr recovery period	AK assay, cytokine secretion, albumin
Treatment **4** – 216 hr (**day 9**)	AK assay, cytokine secretion, albumin
Change medium – 240 hr (**day 10**) - 48 hr recovery period	AK assay, cytokine secretion, albumin
Treatment **5** – 288 hr (**day 12**)	-
Change medium – 312 hr (**day 13**)	AK assay, cytokine secretion
Change medium – 360 hr (**day 15**)	Lipid peroxidation, DNA damage, albumin

### Impact of the selected panel of NMs on human microtissue membrane integrity

**Figure 2:**
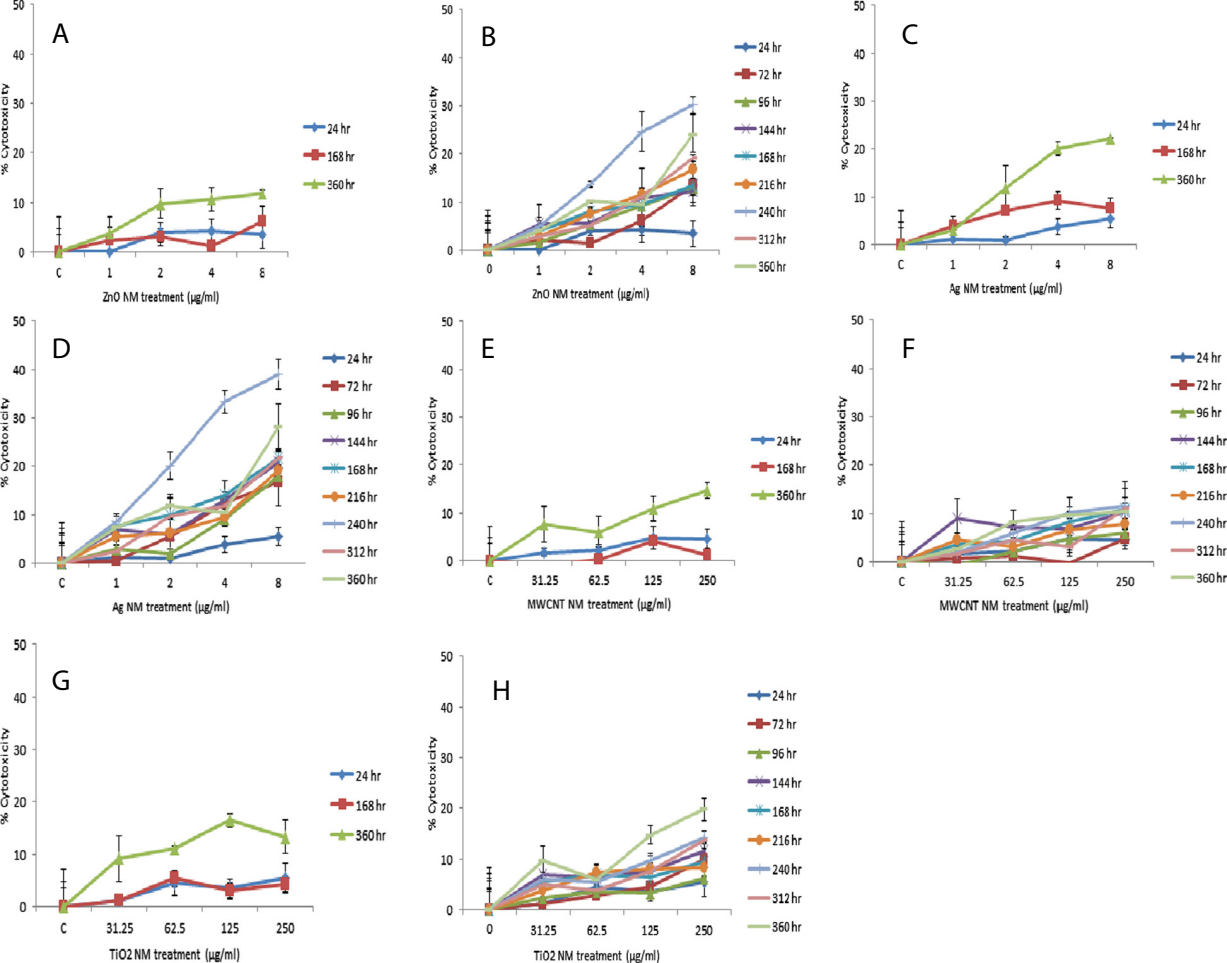
**Cytotoxicity in human liver microtissue following a single or repeated exposures of a panel of engineered nanomaterials.** The microtissue were exposed to cell medium (control)/or NMs over a period of 360 hr as measured by AK release and measured via ToxiLight™ cytotoxicity assay. Values represent mean ± SEM (n = 3). Single exposure **A)** ZnO **C)** Ag **E)** MWCNT **G)** TiO_2_. Repeated exposure (0, 72, 144, 216 and 288 hr) **B)** ZnO **D)** Ag **F)** MWCNT **H)** TiO_2_.

**Figure 3:**
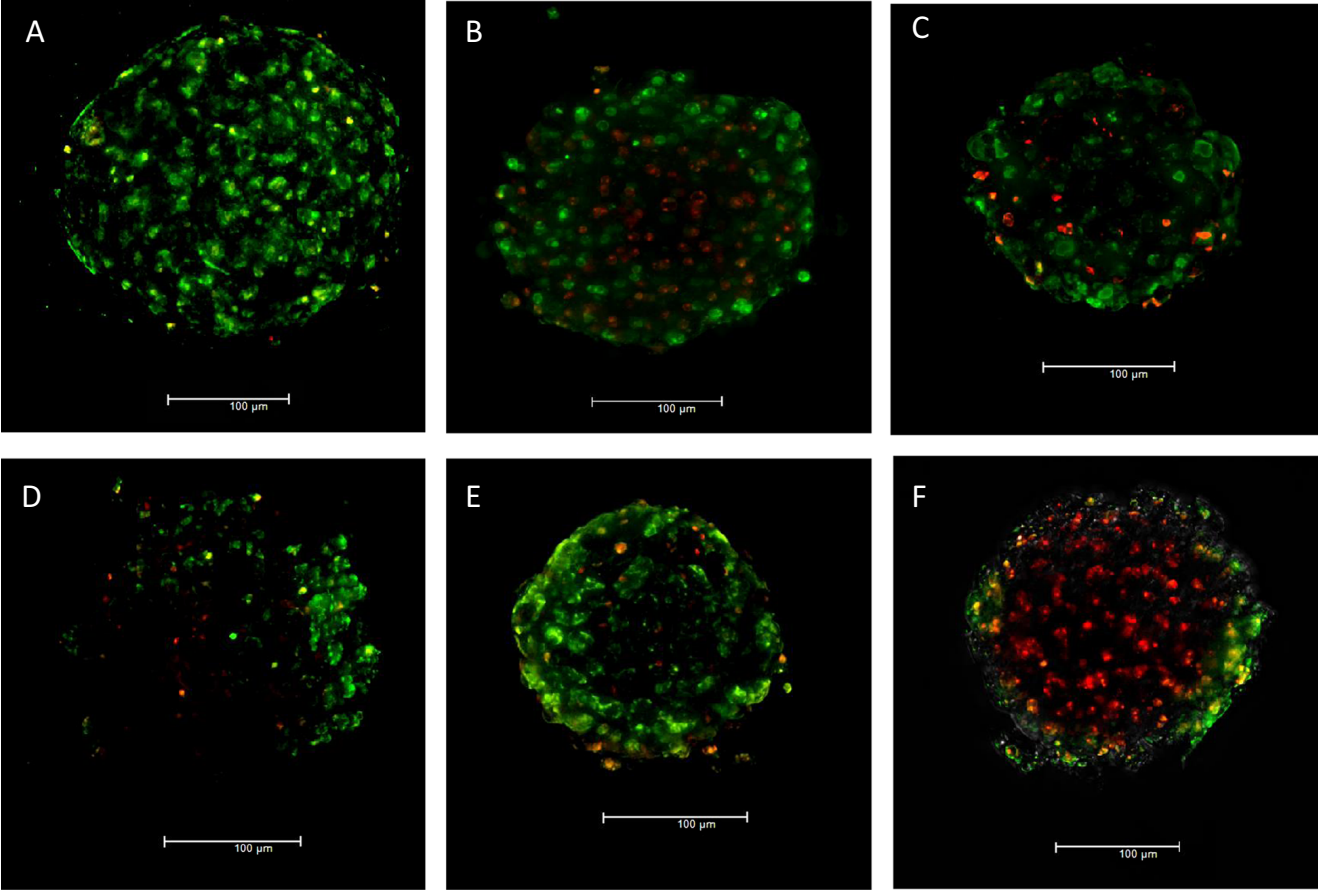
**Live/dead staining of human liver microtissue at 240 hr post repeated exposure. A)** Control **B)** four treatments of ZnO (NM 110) NM at 8 μg/ml **C)** four treatments of Ag (NM 300) NM at 8 μg/ml **D)** four treatments of MWCNT (NM 400) NM at 125 μg/ml **E)** four treatments of TiO_2_ (NRCWE 002) NM at 125 μg/ml **F)** 0.5% Triton-× 100 (24 hr – positive control). As a note the microtissue treated with the MWCNT was partially covered with the NMs hence the quality of the image is inferior to the rest.

From the adenylate kinase (AK) data it was evident that there was a concentration-dependent decrease in cell membrane integrity over time following a single and repeated exposure of the NMs (Figure 2). These effects were more apparent after the repeated exposures although they only became statistically significant for the ZnO and Ag NMs at the later time points (p < 0.05 – 240, 312 and 360 hr exposure) (Figure 2B and D). It is important to note that an LC_50_ was not reached for any of the NMs at the any of the time-points or doses investigated here. Finally, no NM interference was noted with this assay. In addition, live/dead staining of microtissue further supported the AK data depicting increasing cell death following repeated exposure to the panel of nanomaterials over time (Figure 3).

### Cytokine secretion from liver microtissue following treatment with four engineered NMs

Changes in cytokine production as a consequence of NM exposure were assessed within the supernatant of exposed human liver microtissue and quantified via BD™ Cytometric Bead Array cytokine flex sets. The levels of human interleukin (IL) IL1β, IL8, IL10, interferon-γ (IFN-γ) and tumour necrosis factor-α (TNF-α) were measured. IL8 is a chemokine mediating the activation and migration of a wide variety of inflammatory cells including neutrophils into tissue, hence playing a pivotal role in initiation of an inflammatory response [25]. IL10 is a multi-functional cytokine with diverse effects on a wide range of hematopoietic cells. The cytokine’s principle function is to terminate inflammatory responses [26,27]. IL10 also plays a key role in differentiation of regulatory T cells, which figure prominently and importantly in the control of immune responses and developing a tolerant immune state [26].

For the single exposure experiments a concentration dependent increase in IL8 and IL10 was observed. This increase was most evident 24 hr post exposure with these levels dropping over time and returning to background control levels after 360 hr post exposure (Figure 3A and B). Additionally, no significant difference was observed between the NMs. It was discovered that repeated exposure to the NMs resulted in additional secretion of IL8 and IL10 from the tissue at the later time-points although these levels did not reach those of the initial exposure in most instances (24 hr) (Figure 3C and D). Additionally, statistically (p < 0.05) significant differences were found at the latter time-points between the NMs with higher levels of both cytokines following exposure to the Ag and ZnO NMs.

Finally, we noted very small amounts of TNF-α and IFN-γ secreted from the microtissue following repeated exposures of the Ag and NM ZnO NM at the latter time points (data not shown), while no IL1β was detected for any of the NMs, doses or time-points investigated here.

### Lipid peroxidation measurements

**Figure 4:**
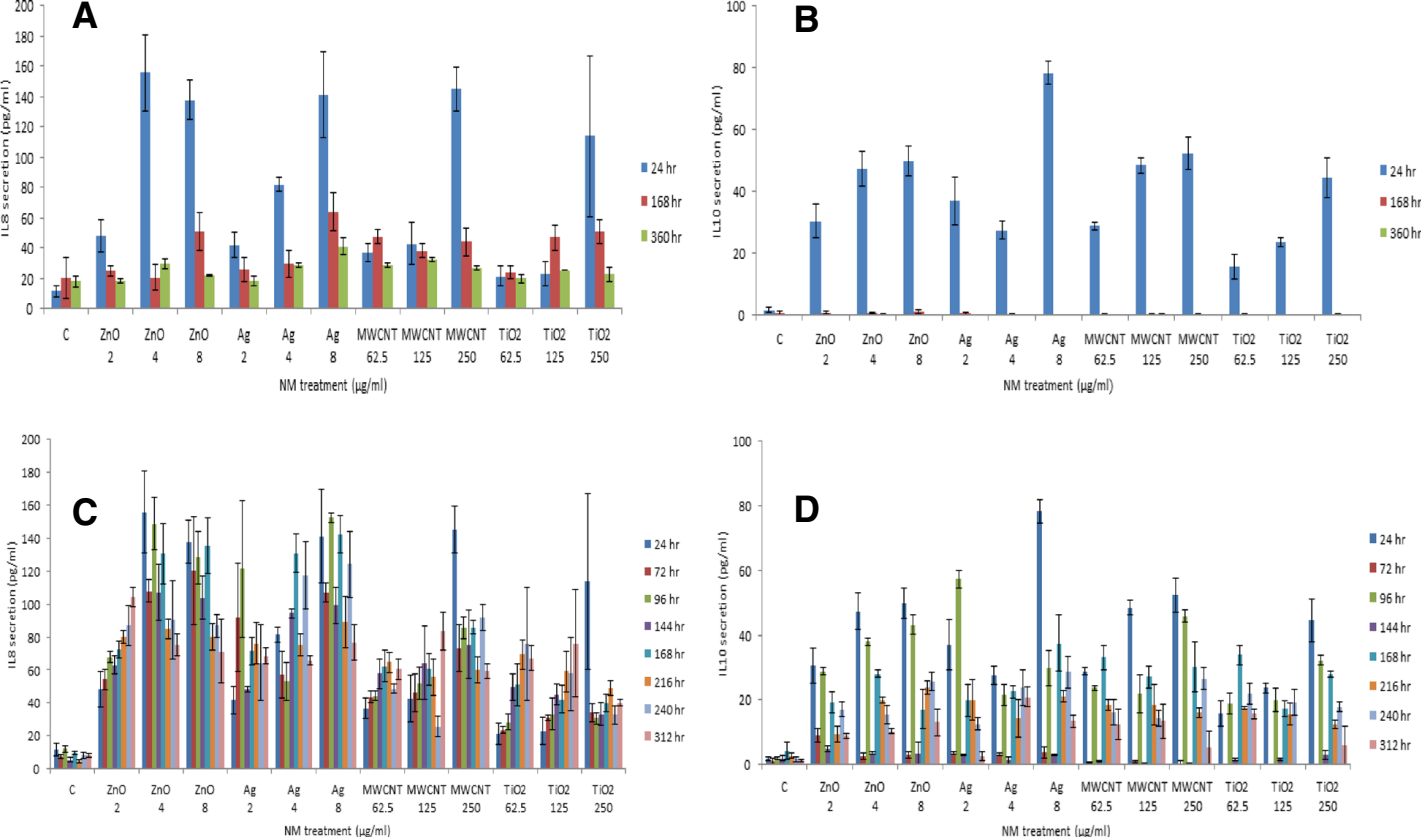
**IL8 and IL10 secretion of human liver microtissue following single and repeated exposure of a panel of engineered NMs.** The tissues were exposed to cell medium control or NMs over a period of 360 hr. Values represent mean ± SEM (n = 3). **A)** IL8 secretion following a single exposure of a panel of engineered NMs **B)** IL10 secretion following a single exposure of a panel of engineered NMs **C)** IL8 secretion following the repeated exposure of a panel of engineered NMs **D)** IL10 secretion following the repeated exposure of a panel of engineered NMs.

Lipid peroxidation which is essential as a means of assessing oxidative stress was quantified following the single or repeated exposures of the four engineered NMs. Interestingly no changes in thiobarbituric acid reactive substances (TBARS) levels were noted following single exposure of any NMs (Figure 4). As a contrast however repeated exposure to the ZnO and Ag NMs at the higher concentrations (ZnO - 4 and 8 μg/ml and Ag - 2, 4 and 8 μg/ml) resulted in significantly higher levels of TBARS from the exposed tissue (Figure 4A and B) while no changes were noted for the MWCNT or TiO_2_ NMs.

### DNA damage

**Figure 5:**
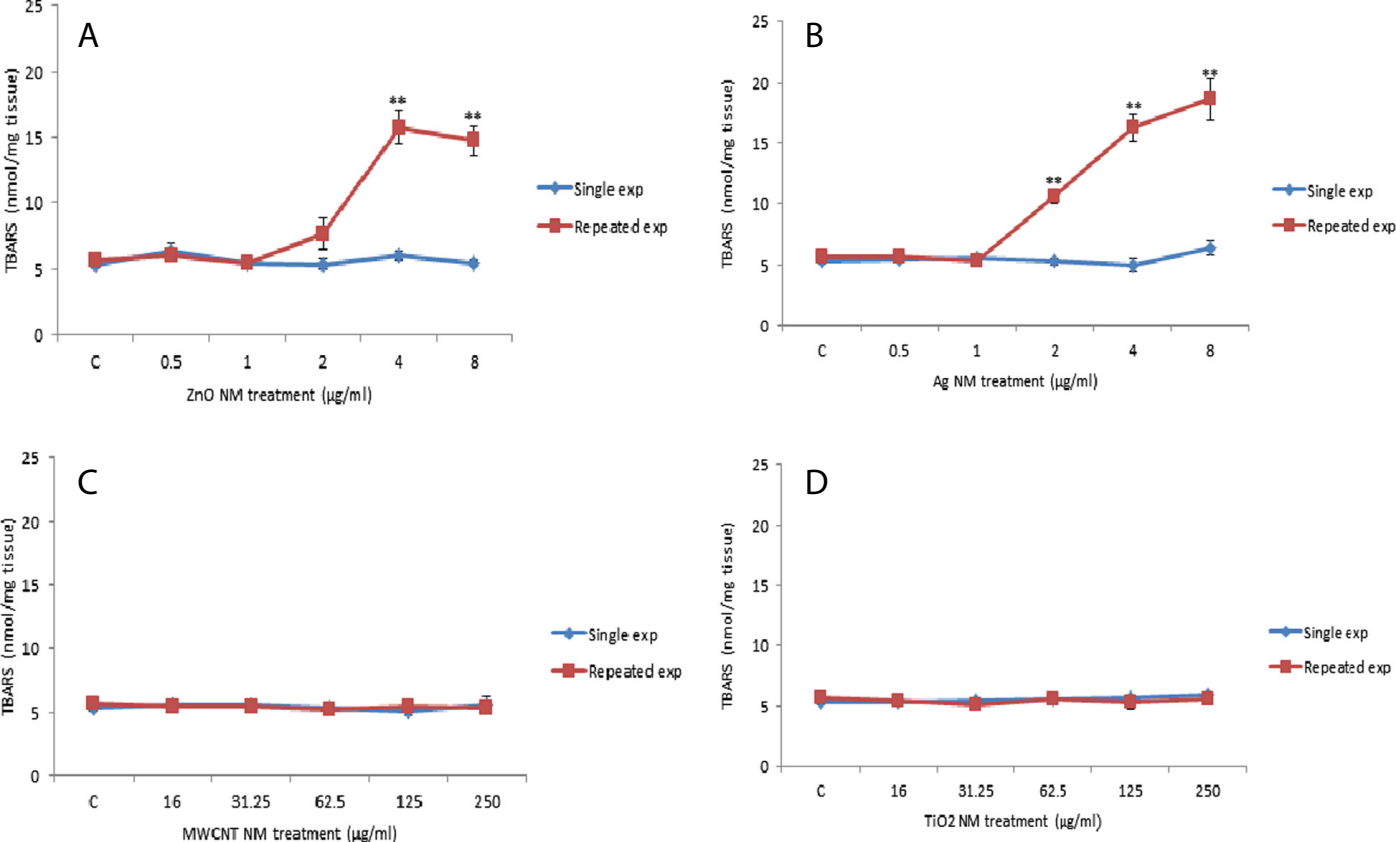
**The effects of increasing concentration of NMs on lipid peroxidation of human liver microtissue.** The tissue was exposed to cell medium (control) or NMs on single or multiple occasions. The data is presented as mean TBARS (nmol/mg tissue) (± SEM) from three experiments (n = 3), significance indicated by * = p < 0.05 and ** = p < 0.005, when NM treatments are compared to the control. **A)** ZnO NMs **B)** Ag NMs **C)** MWCNT NMs **D)** TiO_2_ NMs.

In order to investigate the level of DNA damage caused by a single or repeated exposure of the engineered NMs the comet assay was utilised. A significant increase in DNA strand breaks was noted following exposure to the higher concentrations of all four NMs with the Ag and ZnO being most DNA damaging (Figure 5). In addition, the strand breaks were most evident following the repeated exposure as in comparison to the single exposures. The net level of Fpg sensitive sites (Fpg treatment minus basal level of strand breaks) for the 8 μg/ml exposure was 3.77 ± 1.33% tail DNA (single exposure) and 7.04 ± 1.83% tail DNA (repeated exposures), which were higher than the respective controls (1.32 ± 0.46 and 0.99 ± 0.39, respectively, p < 0.05 significance for repeated exposure). This indicates that the damage witnessed is partially due to oxidative damage to DNA following exposure to these NMs (Figure 5B). The positive control (Ro19-8022 plus white light) had substantially higher levels of Fpg sensitive sites (21.65 ± 1.52% tail DNA) than the liver microtissue.

### Impact of engineered NMs on albumin production by human liver microtissue

**Figure 6:**
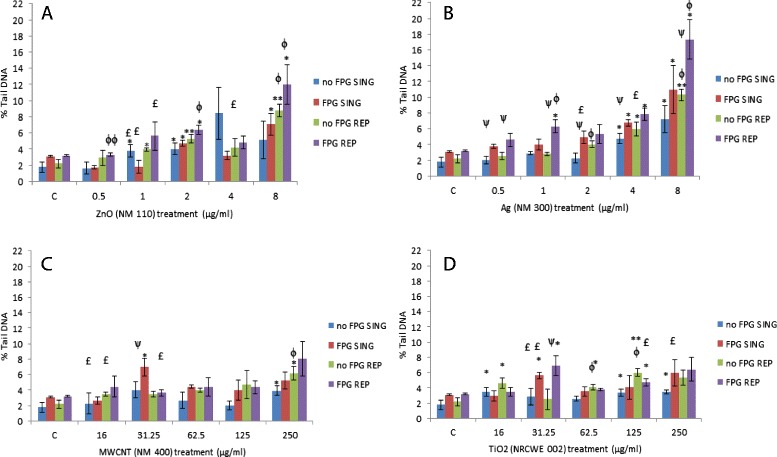
**DNA damage expressed as percentage of tail DNA following exposure of the human liver microtissue to single or repeated exposure of engineered NMs.** The tissues were exposed to cell medium (control) or NMs on one or repeated occasions. Values represent mean ± SEM (n = 3) unless otherwise stated. Significance indicated by * = p < 0.05 and ** = p < 0.005, when material treatments are compared to the control. Ψ = p < 0.05 and ΨΨ p < 0.005 is representative of significant difference between values signifying absence and presence of Fpg enzyme at each given concentration. Φ = p < 0.05 and ΦΦ p < 0.005 is representative of significant difference between single and repeated exposures. £ signifies n = 2 for that particular treatment. **A)** ZnO - NM 110 **B)** Ag – NM 300 **C)** MWCNT - NM 400 **D)** TiO2 – NRCWE 002. SING – single exposure, REP – repeated exposure.

In order to establish whether any of the NMs affected albumin production two concentrations were chosen which were representative of a higher and a low dose for the four materials (0.5 and 4 μg/ml for Ag and ZnO and 16 and 125 μ/ml for the TiO_2_ and MWCNT NMs). There were no significant alterations at any of the investigated time-points following the single exposure of four NMs (Figure 6A). However the repeated exposure of ZnO NM at 4 μg/ml resulted in a significant decrease of albumin production from the liver microtissue at the two latest time-points investigated (Figure 6B). The repeated exposure of three other materials at these concentrations and time-points did not alter albumin production from the liver microtissue in this study.

### CYP3A4 levels

**Figure 7:**
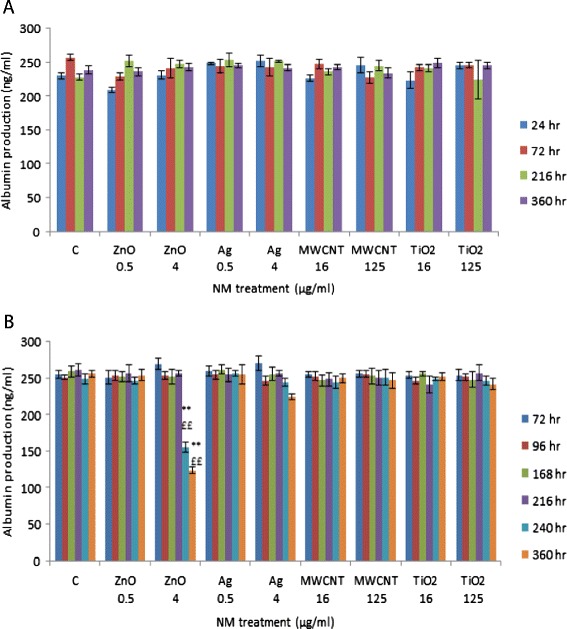
**Albumin production from primary human liver microtissue following exposure to sub-lethal concentrations of the panel of engineered NMs.** The tissue was exposed to medium (control) or NMs over time. The values are representative of mean ± SEM (n = 3), significance indicated by ** = p < 0.005, compared to the control. ££ p < 0.005 represents a significant difference between single and repeated exposures **A)** single exposure **B)** repeated exposure.

**Figure 8:**
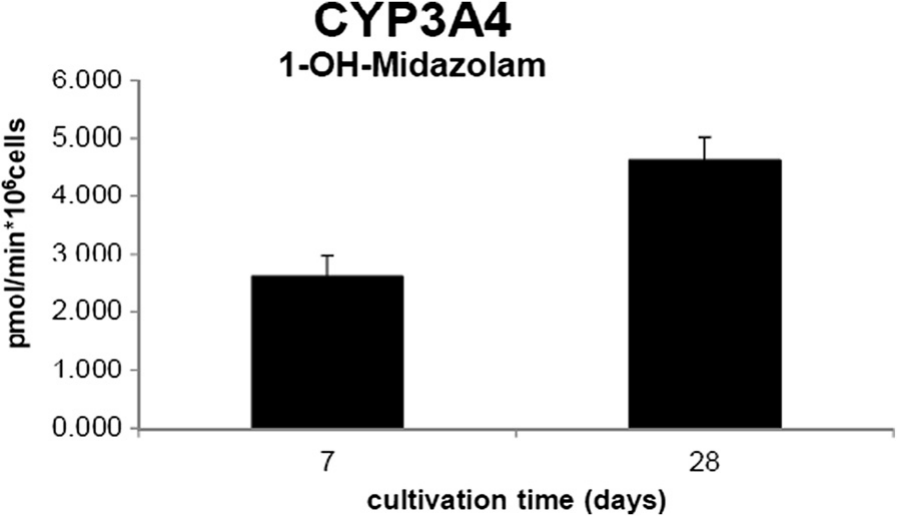
**Basal CYP3A4 activity of 3D human liver microtissue over 28 days in culture.** The liver microtissues were exposed in GravityTRAP™ plates to midazolam for 24 hr. The supernatant from the microtissues were analysed for CYP3A4 mediated midazolam-1-hydroxylation. The values are representative of mean ± SD (n = 3).

Basal hepatic CYP3A4 activity was examined in the liver microtissues over the time span of the experiments (Figure 7). Midazolam was used as an accepted probe substrate to quantify the enzymatic activity of CYP3A4, the main responsible cytochrome for xenobiotic metabolism [28,29]. Interestingly, we found no decrease in the metabolic enzyme activity of CYP3A4 over the entire period of NM treatment, which highlights the suitability of this model system for long-term exposures of nanomaterials Figure 8.

## Discussion

This particular study focused on the toxicological impact of a panel of four engineered nanomaterials on a 3D human liver microtissue model with respect to toxicity, up-regulation of pro/anti-inflammatory mediators, oxidative stress, genotoxicity and hepatic function. This is the first study of its kind that incorporates this new technology in a nanotoxicological context. In this study we demonstrate that the repeated exposure of the NMs is more damaging to the liver tissue as in comparison to a single exposure with the adverse effects more significant following treatment with the Ag and ZnO NMs in terms of cytotoxicity, cytokine secretion, lipid peroxidation and genotoxicity.

Hepatocytes constitute the major cellular compartment of the liver (approximately up to 70% of total liver). These cells are involved in almost all functions that are attributed to the liver. They play a substantial role in the metabolism of exogenous and endogenous lipids and catabolism of blood-derived protein [16]. Hepatocytes manufacture important serum proteins such as complement components and acute phase proteins [16], as well as numerous hormones and cytokines [30-32].

KCs are the resident macrophages of the liver. These cells represent the largest number of macrophages in the mammalian body. KCs eliminate antigens from the circulation and are responsible for the clearance of gut-derived bacteria [16-19]. These macrophages are usually concentrated in the periportal region of the liver, which allows monitoring of the blood entering the organ [16]. It is believed that due to the constant exposure to low levels of gut-derived antigens, KCs are in a permanent semi-activated state. Similarly to other macrophages, once these KCs are fully activated they are capable of aggressive phagocytosis and antigen uptake and the production of an array of mediators involved in protein degradation, cytotoxicity and defence mechanisms [33-35]. KCs are also extremely important in the maintenance of liver tolerance (in which the organ does not mount an immune response to the antigen) [19]. It is understood that following the initial activation and production of a pro-inflammatory response, KCs release IL10 which down-regulates the production pro-inflammatory cytokines [14,19].

SECs (3% of total liver volume) form an important filtration barrier between macromolecules (might also include NMs) and blood cells and hepatocytes – preventing direct contact and influencing the extent of exchange of various substances [16,36].

As already mentioned, nanomaterials entering the blood stream will accumulate within the liver [8,37,38]. In addition, advances in the field of nanomedicine will no doubt result in an increase in direct entry of NMs into the circulatory system. The NMs designed specifically for drug delivery are generally developed to have specific surface properties which influence the fate in the body, including increasing blood circulation time [39,40]. The presence of these NMs in the blood allows distribution to a wide range of target organs, increasing the likelihood of the accumulation of materials in the liver.

In this study NM induced cytotoxicity was evaluated utilizing the ToxiLight™ bioassay kit which measures adenylate kinase release from cells with damaged membranes. AK is an enzyme present in all eukaryotic cells which plays a critical role in intracellular energy homeostasis. From the AK membrane integrity data a concentration dependent decrease in cell membrane damage was noted over time following the single and repeated exposure of the NMs. The prolonged observation time up to 360 hr allowed detection of toxicity after very low single exposures where the usual 3-24 hr assays would show no effect. The adverse effects were more apparent after the repeated exposures, with the toxicity being higher for ZnO and Ag NMs at the later time points. It is important to note that an LC_50_ was not reached for any of the NMs at any of the time-points or doses investigated here. The doses in this study were selected as they represented acute sub-lethal doses from previous studies in a hepatocyte cell line [24] and human primary hepatocytes [31]. It is interesting that multiple exposures to low concentrations of NMs (in particular for the Ag and ZnO NM) resulted in increased cytotoxicity over time which can be attributed to the internalisation and accumulation of materials within the cells. This is extremely important as the accumulation of NMs might account for any potential toxicity that might occur following human exposure scenarios and might be more representative of realistic *in vivo* scenarios and bring up questions about the relevance of acute *in vitro* single cell type toxicity studies at least for the purpose of risk assessment. It also seems appropriate to mention the importance of the *in vitro* dose selection as a representative of realistic *in vivo* exposure [41,42]. In this particular study an *in vitro* dose of 8 μg/ml was the highest dose for ZnO and Ag exposure. Although this dose might be high with regards to the lung burden which might be reached after inhalation exposure [41,43,44], it is a relatively low dose in the context of an *in vitro* study. Having said this, a life time of exposure or IV administration of nano drugs might result in similar doses reaching the liver *in vivo*. Furthermore, it has been shown that *in vitro* studies can be a good indicative of *in vivo* response dependant on the NM and the investigated specific outcome [15,17,45]. It should be noted that we are aware that the repeated dose exposures utilised in this study for the TiO_2_ and MWCNT are high and in all reality would not be reached in *in vivo* settings. However we believed it important to use the same sub-lethal doses in order to allow for the continuation of previous *in vitro* studies. This also permitted for the validation of the new technology in a nanotoxicological context.

Next, IL1β, IL8, IL10, IFN-γ and TNF-α secretion was measured as a means of evaluating NM induced pro/anti-inflammatory response from the human liver microtissue. Firstly, it was discovered that IL8 and IL10 were up-regulated 24 hr post exposure in the single dose experiments with these levels returning to background levels over time. This data could suggest that the pro/anti-inflammatory response in the liver is transient after removal of the exposure despite that the cytotoxicity indicated by AK release appears to increase over time. Interestingly a similar IL10 response pattern was noted from the liver after a low intravenous dose to the same Ag NM in a mouse model [14]. The multiple exposure experiments resulted in secretion of IL8 and IL10 from the tissue at the later time-points, however these levels did not reach those of the initial exposure in most instances (24 hr). Additionally, significant differences were found at the latter time-points between the NMs with higher levels of both cytokines following exposure to the Ag and ZnO NMs most notable at the higher doses. This data might indicate there is a degree of sensitization and IL10 mediated tolerance in the liver over time. Another explanation for this decrease of cytokine release could be cell death over time however as the same general pattern is noted for all four NMs including the two with the very low cytotoxicity this might be unlikely. The cytokine secretion patterns observed in this study suggests that future investigations might benefit on concentrating on the earlier time points post exposure for IL8 and IL10 release. Finally, very little up-regulation of TNF-α, IFN-γ or IL1β was noted from the microtissue following any of the exposure regiments at the concentrations and times investigated within the parameters of this study. The lack of a TNF-α response is slightly surprising as the same Ag NM induced an up-regulation of the cytokine both in a mouse intravenous exposure model [14] and an *ex vivo* rat hepatocyte and KC co-culture model [Filippi C, Kermanizadeh A, Stone V: Kupffer cell contribution to the hepatic response to nanomaterials. In preparation].

In order to establish if a single or multiple exposure to the panel of NMs induced oxidative stress in the microtissue the levels of lipid peroxidation products were determined with an assay that detects reactive carbonyl compounds such as malondialdehyde. Lipid peroxidation measurement (oxidative degradation of lipids) is a widely accepted tool of assessing oxidative stress. Malondialdehyde and 4-hydroxynonenal are secondary bi-products, generated by degradation of lipid hydroperoxides from the lipid membranes. Once again the single exposure to any of the NMs did not induce a significant oxidation of cell membrane lipids. These findings provided corroborating evidence to the single exposure toxicity and inflammation data discussed above. As a contrast however, repeated exposure to the ZnO and Ag NMs at the higher concentrations resulted in lipid peroxidation in the exposed microtissue. These findings seem to suggest that there is some degree of damage to the tissue following the five treatments of the two NMs at the higher concentrations with oxidative stress being one of the contributors to these adverse effects (lipid peroxidation was only measured after five treatments (360 hr)). Previous studies have shown that NMs are able to induce lipid peroxidation in hepatocytes as demonstrated in an experiment with 3 nm Ag NMs which induced a two fold increase in malondialdehyde in HepG2 cells at a dose of 25 μg/ml [46] or repeated exposure in liver of Medaka fish after 14 days of treatment [47].

We then investigated genotoxic effects following the sub-lethal exposure of the four NMs. In this study the comet assay was utilised as it requires relatively little sample material and it has become a standard tool in genetic toxicology of particulate matter [48]. A significant increase in strand breaks was noted following exposure to the higher concentrations of all four NMs with the Ag and ZnO being potent at induction of these breaks. Similar findings were noted in previous experiments for these NMs in C3A cells following a 24 hr exposure [49]. The strand breaks were most evident following the repeated exposure in comparison to the single exposures. In addition, there was increased levels of Fpg sensitive sites, indicating that the damage is partially due to oxidative damage to DNA following exposure to Ag NMs. Overall this data suggests that the repeated exposure to these NMs (especially the highest concentrations) does indeed induce genotoxicity albeit at fairly low levels however due to some technical issues (to be discussed in the following sections) no further conclusions can be made on the findings from the assay.

Albumin is by far the most abundantly produced protein from the liver and therefore an excellent candidate for the quantification of normal liver function. Here we demonstrate that with the exception of repeated treatments of ZnO NM (4 μg/ml – 240 and 360 hr) no change was noted in albumin production after any of treatments within this study. We have shown previously that exposure of C3A cells to NMs resulted in no change in the levels of albumin with the exception of the ZnO NM at LC_50_ concentration [24]. In addition a 24 hr intravenous exposure (24 μg/animal) of Ag NM also did not result in a significant change in the levels of albumin production compared to the control animals [14]. This data suggests that despite of varying degrees of cell death, with the exception of the ZnO NM after repeated higher level exposure the NMs do not affect hepatocyte function *in vitro* in terms of albumin production. The inhibition of albumin production by the ZnO NMs occurred late after repeated exposure when cytotoxicity and lipid peroxidation were overt which might provide an explanation, although similar toxicity signs of repeated Ag exposure gave no reduction in albumin production. Albumin is a very important transport protein for Zn ions in the blood. Another theory for the observed findings is that the ZnO NMs/Zn^2+^ are binding the protein and interfering with the assay, although this does not explain why the effects are only visible at the latter time points.

The cytochrome P450 superfamily are a number of enzymes that are involved in a diverse array of substrate specific and catalytic activities [28]. The CYP450 enzymes and their co-factors are embedded in the membrane of the endoplasmic reticulum and play a crucial role in the metabolism of drugs, chemicals and endogenous substrates in the liver [50]. Due to their importance in healthy functioning hepatocytes this superfamily is usually investigated as markers for normal liver function [51]. Here we found no decrease in metabolic enzyme CYP3A4 over a period of 21 days. In addition, these CYP3A4 levels are comparable to primary hepatocytes.

The use of *in vitro* hepatocyte models have been beneficial for last two decades in research and various application areas. Traditionally, primary hepatocytes appear as the closest model for the human liver. However, these cells are typically phenotypically unstable, and have an extremely short life span. In addition there are significant variations between different human donors [52,53]. Hepatocyte cell lines on the other hand are cheap and much easier to maintain. Hence they are often utilised as surrogates for human primary hepatocytes. However their use in absorption, metabolism, excretion and toxicity studies have been heavily questioned as the basal levels of their metabolizing enzymes is greatly reduced compared to primary cells [54]. In addition, most *in vitro* or *ex vivo* nanotoxicological hepatic studies are carried out utilizing hepatocyte cell lines or primary cultures lacking any non-parenchymal cells (i.e. KC or SECs). As expressed above these cells are crucial in terms of the whole organ response to NMs. In order to address all of above issues in this study we have used the InSphero 3D human microtissue. This model offers a stable and functionally active system that has some notable and extremely important advantages over the traditional *in vitro* hepatic models [22]. Additionally, this model allows the use of primary human hepatic cells. This is crucial as most primary hepatocyte data generated up to date have utilised cells from rodents. These inter-species differences could be important both in terms of nanotoxicology but also in the future experiments involving NM exploitation for the development of field of nanomedicine. The liver cells in this model are viable for long periods of time which allows for multiple treatments of NMs over period of weeks which might be more representative of potential exposure scenarios, *in vivo* toxicity and disease progression. In addition the hepatocytes in the 3D microtissue model maintain high levels of the metabolic enzymes for their entire life-span. As already touched upon, this model also incorporates the most important hepatic cell populations at the approximate percentages as would be found in a healthy liver [22]. Additionally, it has been demonstrated that there is little variability between individual plates. Finally, once established this model could significantly reduce the need for animal testing.

Despite the numerous advantages of the InSphero 3D human liver microtissue which have been outlined and discussed above, there are also some limitations to this technology which need to be taken into account that are important both in the context of this study and any future experimentations. The commercially available model system requires to be purchased and therefore the price needs to be taken into account in any study design. In addition, due to the limed volumes of supernatant there are some restrictions to the assays which can be utilised. On the same note due to the same quantity issues there is very little room for error. Finally, for assays which require individual cells (i.e. the comet assay) an efficient homogenization method is required. In this study this was a problem which is highlighted in Figure 5, where some samples did not contain the required cell numbers hence some replicates are missing. Thus, further optimization of the homogenization protocol may be necessary. Finally, there is a possibility that due to the lack of blood flow and 3D spheroid structure not all the inner cells in the microtissue might directly encounter the NMs. However, since the cytotoxicity was not only restricted to the outer layer and was distributed throughout the sphere, it can be argued that some NMs do penetrate to the central regions of the microtissue.

## Conclusions

With the advances in the fields of nanotechnology and nanomedicine, the potential for public and occupational exposure is likely to increase, so that there is an urgent need to consider any potential health consequences associated with this increased exposure to nanomaterials. The potential for NM translocation to distal organs following a variety of exposure routes is a realistic prospect, with the liver accumulating a large proportion of the total or translocated dose. Although advances have been made in identifying the potential nanotoxicological effects on the liver, there are still large gaps in the knowledge currently available. Therefore, more information is required for the likely exposure routes and levels, combined with a mechanistic understanding of the fate and behaviour of the NMs to determine the biological activity of the nanomaterial in question [55]. As a means of better understanding the mechanistics of NM mediated hepatotoxicity, we believe that the 3D human liver microtissues utilised within this study are an extremely strong candidate for further progression of *in vitro* hepatic nanotoxicology.

## Methods

### Nanomaterials

Nanomaterials were purchased as stated: NM 110 (BASF Z-Cote; ZnO, uncoated, 100 nm), NM 300 (RAS GmbH; Ag capped with polyoxylaurat Tween 20 - <20 nm), NM 400 (Nanocyl; entangled MWCNT, diameter 30 nm). All NMs were sub-sampled under Good Laboratory Practice conditions and preserved under argon in the dark until use. The NRCWE sample was procured by the National Research Centre for the Working Environment (Copenhagen, Denmark). The NRCWE 002 NM was produced from the NRCWE 001 (TiO_2_ rutile 10 nm) as previously described [24].

### Characterisation of the panel of engineered nanomaterials

Investigated nanomaterials were characterised by a combination of analytical techniques in order to infer primary physical and chemical properties. Phase compositions and average crystallites sizes were determined from powder X-ray diffractograms obtained at room temperature (25°C) using a Bruker D8 Advanced diffractometer in reflection mode with Bragg-Brentano geometry. A sealed Cu X-ray tube was run at 40 kV and 40 mA, wavelength Cu K_α1_ 1.5406 Å from a primary beam Ge monochromator, fixed divergence slit 0.2°, step size 0.02, step time 1 s step^-1^, linear PSD detector (Lynx-eye) with opening angle 3.3°. The sample holders used for the reflection data were either a standard sample holder containing an approximately 2 mm thick sample or a single Si sample holder. The Ag sample was measured as transmission in a capillary. The phases were identified by using the EVA 14.0 software from Bruker AXS. The ratios and size were calculated using Topas 4.1 from Bruker. The primary and aggregate size range, shape and crystal structure of the test materials were determined by 3010 transmission electron microscope operating at 300 kV (Jeol, Japan). Surface areas and pore volumes were obtained by nitrogen adsorption on a Micrometritics ASAP2000 Accelerated Surface Area and Porosimetry System at an adsorption temperature of -196°C, after pre-treating the sample under high vacuum at 300°C for 2 hr. In order to assess the dissolution of ZnO and Ag, the materials were diluted in both ultrapure and filtered water and complete medium. The analysis was carried out using an atomic absorption spectrometer (Ag and Zn Hollow cathode lamp) (Perkin Elmer AAnalyst 200, USA).

A summarised list of the measured physical and chemical properties of the selected NMs has been reproduced from previously described work [24] (Table 1). Furthermore the hydrodynamic size distributions of the NMs dispersed in 3D InSight™ human liver maintenance medium (InSphero, Switzerland) were determined in the 5-20 μg/ml concentration range by Nanoparticle Tracking Analysis (Nanosight LM20, USA) (Table 1).

### Morphological characterization of 3D human liver microtissues

The primary human liver cells were obtained from Bioreclamation IVT (Brussels, Belgium). In all instances, consent was obtained from all donors. For ethical and privacy reasons no information was provided which might identify the donor. For characterisation purposes the microtissues (Hepatocyte lot IZT; Non Parenchymal Cell lot JJB) were harvested after 7 (day 0 – first NM treatment) and 28 days (21 days after first NM treatment) culture time into 1.5 ml tubes, washed with 1 ml PBS and fixated with 4% PFA for 2 hr at room temperature. The specimens were over-layed with agarose and further processed for paraffin embedding and histological staining. Next, paraffin sections were stained with H&E. Immunohistochmistry staining for KCs was performed with a CD68 antibody (Novocastra Laboratories Ltd, USA).

### Microtissue maintenance and NM treatment

The human liver microtissue was maintained in human liver maintenance medium at 37C, 5% CO_2_. The treatment regiments and the different end points investigated have been outlined in Table 2.

The Ag NM was supplied in de-ionised water (85%) with 7% stabilizing agent (ammonium nitrate) and 8% emulsifiers (4% each of Polyoxyethylene Glycerol Trioleate and Tween 20). All other materials were supplied as dry powders. The NMs were dispersed in cell culture grade water with 2% FCS. The nanomaterials were sonicated for 16 mins without pause following instructions in the protocol developed for the EU funded project – ENPRA [56]. Following the sonication step, all samples were immediately transferred to ice before being diluted in medium just prior to the experiments. In this study we have utilised five concentrations for all NMs which included 16, 31.25, 62.5, 125 and 250 μg/ml for the TiO_2_ (NRCWE 002) and MWCNT (NM 400) and 0.5, 1, 2, 4 and 8 μg/ml for the ZnO (NM 110) and Ag (NM 300) which were considered as non-lethal concentrations to hepatocytes based on previous studies [24,31]. In this study all concentrations are expressed as μg/ml. The main reason for this is that the microtissues are clustered as spheroids and expressing the treatments as μg/cm^2^ would not be very relevant or informative. The aim of the repeated exposure experiments was to assess the adverse effects that might be attributed to the increased internalisation and accumulation of materials within the cells over time.

### Adenylate kinase assay

The loss of cell membrane integrity was evaluated utilising a ToxiLight™ bioassay kit (Lonza, USA). Briefly, 20 μl of cell supernatant was transferred to a luminescence compatible plate before the addition of 80 μl of AK detection buffer. The plates were incubated for 5 mins at room temperature before the luminescence was measured. These experiments included the appropriate controls which included tissue treated with medium or ToxiLight™ 100% lysis reagent.

### Live/dead staining

In order to get pictorial representations of NM induced mirotissue cell toxicity a live/death cell staining kit (Abcam, USA) was utilised. The kit uses a cell permeable green fluorescent dye (Ex/Em - 488/518 nm) to stain live cells, while dead cells are stained via propidium iodide (Ex/Em – 488/615 nm). Briefly, microtissue was transferred to 8 well microscopy chambers (Ibidi, Germany). A volume of 200 μl of the staining solution was added to all samples before incubation for 15 mins at 37°C. The tissue was then observed under a Leica AF6000 inverted wide field fluorescence microscope (Leica, Germany). In all instances images were captured as a z-stack before undergoing a 3D de-convolution and being represented as a final 3D projection.

### Cytokine secretion

The levels of human IL1β, IL8, IL10, IFN-γ and TNF-α secreted from the microtissue was determined in the cell supernatant using the BD™ Cytometric Bead Array cytokine flex sets (bead based immunoassay; BD Biosciences, USA).

Flow cytometry was used to discriminate between different bead populations based on size and fluorescence, according to the manufacturer’s instructions. The flex sets employ micro particles with discrete fluorescence intensities to detect soluble analytes (in this case cytokines).

### Lipid peroxidation

NM induced oxidative stress (in form of TBARS) was quantified via a lipid peroxidation kit (Abcam, USA). Briefly, the microtissue was homogenized on ice in 300 μl of lysis buffer and centrifuged at 13000 g for 10 mins. This was followed by the addition of 600 μl of thiobarbituric acid and an incubation of 95°C for 60 mins. The samples were cooled on ice for 10 mins and the fluorescence measured at Ex/Em 520/538 nm. The fluorescence signal was compared to a standard curve of malondialdehyde (MDA).

### Detection of DNA damage in exposed liver microtissue

The Fpg (formamidopyrimidine [fapy] – DNA glycosylase) modified comet assay was utilised to measure DNA strand breaks and specific oxidatively damaged DNA such as 7,8-dihydro-8-oxoguanine and ring-opened, 2,6-diamino-4-hydroxy-5-formamidopyrimidine and 4,6-diamino-5-formamidopyrimidine products, based on the method described previously [57].

In these experiments two pieces of microtissue were homogenised, before a mixture of cells and 0.5% low melting point agarose was added to Gel Bond® films (Lonza, USA). Following 10 mins of solidification, cells were lysed overnight at 4°C in lysis buffer (2.5 M NaCl, 100 mM EDTA, 10 mM Tris-base, pH 10, containing 10% DMSO and 1% Triton X-100). The films were washed three times for 5 mins with Fpg-enzyme buffer (40 mM HEPES, 100 mM KCl, 0.5 mM EDTA, 0.2 mg/ml BSA - pH 8), covered with 100 μl of either buffer or Fpg (Fpg enzyme was a gift from Professor Andrew Collins (University of Oslo, Norway) in buffer (1:30), sealed with a cover slip and incubated for 30 mins at 37°C. Fpg cleaves DNA at locations of oxidation leading to a greater tail for cells exhibiting oxidative DNA damage [58]. The films were then transferred into chilled electrophoresis tanks. After alkaline unwinding (pH 13) for 20 mins, electrophoresis was performed for 10 mins at 270 mA, 24 V. The slides were neutralized three times for 5 mins using a neutralization buffer (0.4 M TrisBase, pH 7.5). Before the final count the films were dried and stained with GelRed (2 in 10000 dilution, 40 μl per slide). The percentage of DNA in the tail was measured using an automatic image analyser (Comet Assay IV; Perceptive Instruments, UK) connected to a fluorescence microscope. The images were captured using a stingray (F-033B/C) black and white video camera. In all instances a total of 50 cells were analysed per gel with the mean used as the experimental unit in the statistical analysis. A reference control sample was utilised in each experiments (corresponding to each electrophoresis) that included undamaged or photosensitizer Ro19-8022 and white light exposed peripheral mononuclear blood cells, which generates Fpg sensitive sites. The Ro19-8022 was a kind gift from F. Hoffmann (Roche, Switzerland).

### Albumin production

After exposure the supernatants (from both the control and treated cells as described above) were collected and stored at -80°C. The supernatants were centrifuged at 1000 g, were diluted two fold and albumin levels determined by ELISA according to the manufacturer’s instructions (Bethyl laboratories, USA).

### CYP3A4 measurements

3D InSight™ human liver microtissues were incubated in medium containing midazolam as a probe substrate for CYP3A4 activity. The amount of midazolam-1’-hydroxylation was assessed after 24 hr incubation at culture days 7 and 28. For all time points three replicates single microtissues were analysed for each time point. After the 24 hr incubation period with midazolam, the supernatants were harvested and stored at -80°C. The quantification of midazolam-1-hydroxylation in cell culture supernatants was performed by Pharmacelsus® (GmbH, Germany).

### Statistical analysis

All data are expressed as mean ± standard error of the mean (SEM). For statistical analysis, the experimental results were compared to their corresponding control values using full-factorial ANOVA with Tukey’s multiple comparison. All statistical analysis was carried out utilizing Minitab 17. A p value of <0.05 was considered to be significant. All experiments were repeated a minimum of three times unless otherwise stated.
